# The Pennsylvania Journal of Dental Science

**Published:** 1877-03

**Authors:** 


### Editorial

### THE PENNSYLVANIA JOURNAL OF DENTAL SCIENCE.

In the last issue of this journal the editor says: "The present number of the *Journal* completes the third volume, and in all probability the career of the *Pennsylvania Journal of Dental Science*."

We regret that circumstances have rendered the suspension of this journal necessary, We have always read it with interest, and have regarded it as one of the outspoken independent journals, and we regret to lose it from our list of exchanges. We hope that though this medium of communication has ceased we will, not be deprived of contributions from Dr. Welchens' pen. The Dr. is a elear and vigorous writer, one who, in justice to himself or the profession, can not afford to be still, or hide his light under a bushel.

The pages of the REGISTER will be open for him whenever he chooses to use them, and we hope that may be often, we will not soon forget the *Pennsylvania Journal of Dental Science.*

